# Transcriptome analysis reveals mRNAs and long non-coding RNAs associated with fecundity in the hypothalamus of high-and low-fecundity goat

**DOI:** 10.3389/fvets.2023.1145594

**Published:** 2023-03-28

**Authors:** Biwei Hou, Min Mao, Shucan Dong, Ming Deng, Baoli Sun, Yongqing Guo, Yaokun Li, Dewu Liu, Guangbin Liu

**Affiliations:** College of Animal Science, South China Agricultural University, Guangzhou, China

**Keywords:** Chuanzhong goat, hypothalamus, fecundity, mRNA, lncRNA

## Abstract

As an important organ that coordinates the neuroendocrine system, the hypothalamus synthesizes and secretes reproductive hormones that act on the goat organism, thereby precisely regulating follicular development and reproductive processes in goats. However, it is still elusive to explore the mechanism of hypothalamic effects on goat fertility alone. Therefore, RNA-seq was used to analyze the gene expression in hypothalamic tissues of goats in high fertility group (HFG: litter size per litter ≥2) and low fertility group (LFG: litter size per litter = 1), and identified the differential lncRNAs and mRNAs and their associated pathways related to their fertility. The results showed that a total of 23 lncRNAs and 57 mRNAs were differentially expressed in the hypothalamic tissue of high and low fertility goats. GO terms and KEGG functional annotation suggest that DE lncRNAs and DE mRNAs were significantly enriched in hormone-related pathways regulating ovarian development, hormone synthesis and secretion, regulation of reproductive processes, Estrogen signaling pathway, Oxytocin signaling pathway and GnRH signaling pathway. And we constructed a co-expression network of lncRNAs and target genes, and identified reproduction-related genes such as NMUR2, FEZF1, and WT1. The sequencing results of the hypothalamic transcriptome have broadened our understanding of lncRNA and mRNA in goat hypothalamic tissue and provided some new insights into the molecular mechanisms of follicle development and regulation of its fertility in goats.

## 1. Introduction

The goat (Capra hircus) is one of the major livestock species, which is widely distributed in various regions due to its roughage resistance and strong adaptability. At the same time, it can provide people with daily necessities such as meat and milk, so it plays an important role in the animal husbandry (1). Fecundity is the ability of animals to produce live offspring, and high fecundity usually means higher economic efficiency in livestock species (2). Kidding trait is an important economic trait in goat reproduction, and kidding number is a key indicator of goat kidding trait, which is directly related to the economic benefits of goat farming (3). In addition, the number of lambs is not only affected by nutrient levels and environment, but also by a complex genetic component of quantitative traits controlled by intrinsically micro-effective polygenes (4). Therefore, improving goat fecundity is the key way to change the status quo of the low level of goat breeding, and it is crucial to study its internal reproductive mechanism in depth.

Long non-coding RNAs (lncRNAs) are transcribed under the direction of RNA polymerase II/III and are longer than 200 nucleotides long-stranded RNAs, which are widely found in various eukaryotic organisms (5). Since lncRNAs do not encode any proteins, and most of the primary structures of lncRNAs are poorly conserved among different species, lncRNAs are generally considered by the scientific community as transcriptional “noise” (6). However, with the in-depth study of the genomic information of organisms, more and more scholars have paid attention to the special functions of lncRNAs in organisms. Studies have shown that lncRNAs play important regulatory roles in epigenetic control and regulation of transcription (7), translation (8), RNA metabolism, as well as in stem cell maintenance and differentiation (9), cell autophagy and apoptosis, and embryonic development (10). Recently, lncRNAs have been identified as an important RNA that regulates fertility-related RNAs in the sheep hypothalamus (11). Chen et al. (4) found that lncRNA LINC-676 and WNT3-AS acting in cis on DRD2 and WNT9B may induce gonadotropin-releasing hormone (GnRH) secretion at different stages by collecting hypothalamic tissues from wild-type and FecB genotyped sheep and performing whole transcriptome sequencing in ewes in follicular and luteal phases. However, there are few studies on mRNAs and long non-coding RNAs associated with fertility in the hypothalamus of high-and low-fecundity goats.

Early studies have shown that there is a high genetic correlation between ovulation rate and kidding rate (12). As the ovulation activity of animals is affected by the reproductive endocrine regulation (13), and the hypothalamus-pituitary-ovary (Hypothalamus-Pituitary-Ovary, HPO) axis plays a leading role in its reproductive regulation, the reproduction-related hormones released by various levels of the HPO axis participate in the whole regulation process as the key signal carrier (14). Where hypothalamic activation and gonadotropin-releasing hormone (GnRH) secretion are considered to be the decisive upstream factors in initiating the reproductive cascade to initiate pituitary and ovarian function. The hypothalamus regulates a large number of reproductive activities by integrating endocrine signals from the whole body and neural signals from the upstream brain, and its neuronal population is the central regulator of energy homeostasis and reproductive function (15). The hypothalamus secretes gonadotropin-releasing hormone (GnRH), which reaches the pituitary gland *via* the pituitary portal system and binds to the corresponding receptors in the adenohypopituitary cells, stimulating them to secrete follicle stimulating hormone (FSH) or luteinizing hormone (LH), etc., to regulate reproductive activity downstream of the body (16). It has been shown that differential expression of hypothalamic genes can have an effect on reproductive traits in animals. Studies indicated that differential expression of the DRD1 gene in the hypothalamus may affect reproductive hormone secretion and ovulation numbers in ewes throughout the breeding season (17). In addition, DRD1 gene knockout in mice also revealed that DRD1 gene influences reproductive activity by regulating the secretion of GnRH, FSH, LH and PRL, suggesting that the differential expression of DRD1 gene in hypothalamus is closely related to the fertility of animals (18).

So far, the most intensive research on lncRNAs has focused on the level of ovarian tissue in the HPO axis, but little is known about its role in the hypothalamus and its mechanism of regulating reproductive hormones in goats, and the synergistic action of hypothalamus, pituitary and ovary can promote the moderate development of sexual organs and the formation of gametes (19). Previous studies have confirmed that the expression levels of certain genes in the hypothalamus directly or indirectly significantly affect reproductive activities such as follicle formation, follicular development and ovulation, while the ovulation rate of goats directly affects the fecundity of goats (20). Therefore, in this study we performed differential expression analysis and gene function analysis using RNA-seq, and established an lncRNA-mRNA interaction network, in order that the correlation between the level of fertility and the expression of certain genes in the hypothalamus of goats could be explored in depth, thus laying the foundation for future studies on the molecular mechanisms regulating fertility in goats.

## 2. Materials and methods

### 2.1. Animals and sample collection

We selected 11 healthy Chuanzhong (CZ) female goats (3.5–4.5 years old) from a commercial farm with more than 3 litters were divided into high fecundity group and low fecundity group. The litter size of female goats in high fecundity group (*n* = 6) were more than 2 per litter, the litter size of female goats in low fecundity group (*n* = 5) were only 1 per litter. These ewes eat and drink freely under natural light conditions. After estrus synchronization, the selected goats were slaughtered and dissected, and the hypothalamus tissues were collected immediately and frozen in liquid nitrogen before being returned to the laboratory for RNA extraction.

### 2.2. RNA extraction and quality determination

Total RNA was extracted from the hypothalamus tissues of 11 CZ female goats using TRIzol reagent (Invitrogen, Carlsbad, CA, USA). The RNA quality was estimated using an Agilent 2100 Bioanalyzer (Agilent Technologies, Palo Alto, CA, USA) and NanoDrop spectrophotometer (ND-2000, Thermo Fisher Scientific, Wilmington, DE, USA). The RNA integrity was evaluated by 1% agarose gel; purified RNA was stored at −80 °C. High quality RNA (quantity >6 μg, concentration ≥200 ng/mL, 1.8 < OD260/280 <2.2, and RNA integrity number >8.5) was used for preparing the cDNA libraries.

### 2.3. RNA library construction and sequencing

The ribosomal RNAs (rRNAs) were removed from the total RNA using the Ribo-Zero rRNA Removal Kit (Illumina, Inc.), and then the RNA was fragmented approximately 200–300 bp. The first-strand cDNA was synthesized using a random hexamer primer and reverse transcriptase. Using the first-strand cDNA as a template, the second-strand cDNA synthesis was subsequently performed. Then, the further hybridization was ligated with Sequencing Adaptor after adenylation of 3′ ends of the DNA fragments. To select cDNA fragment preferentially 300–400 bp in length, the library fragments were purified with the AMPure XP system (Bechman Coulter, CA, USA). Subsequently, PCR amplification was performed to enrich cDNA libraries. The library quality was accessed on the Thermo Scientific StepOnePlus Real-Time System (Thermo Scientific, NY, USA). Finally, the cDNA libraries were sequenced on a Hiseq platform (Illumina Hiseq X-ten PE150) in Shanghai Personal Biotechnology Cp. Ltd. (Shanghai, China), and 150 bp paired-end reads were generated.

### 2.4. Quality control, mapping, and transcriptome assembly

After converting the raw image data generated by the Hiseq platform (Illumina) into raw data in the FASTQ format, quality control was carried out. The clean data (clean reads) were obtained by removing the reads containing adapter or poly-N and other low-quality reads from the raw data using Cutadapt. Swine reference genome and gene model annotation files were downloaded from the genome website directly (asia.ensembl.org/index.html). Then, the clean reads were mapped to the goat reference genome using TopHat2 software. The transcripts obtained were assembled and the abundance estimation was performed.

### 2.5. Identification of lncRNAs

To reduce the false-positive rates, transcripts were assembled to obtain candidate lncRNAs by following steps: (1) Transcripts with a single exon and <200 bp in length were removed; (2) The transcripts with reads coverage <3 were removed; (3) Transcripts with protein coding potency were removed by three software: Coding Potential Calculator 0.9r2 (CPC), Coding Non-Coding Index v2 (CNCI) and Pfamscan (1.6). Subsequently, the number of candidate lncRNAs was obtained.

### 2.6. Differential expression analysis of lncRNAs and mRNAs

LncRNA and mRNA expression in each sample was evaluated based on the fragments per kilobase per million mapped reads (FPKM). Those lncRNAs and mRNAs with |log2(Fold change) | > 1 and significant *P*-value < 0.05 were considered as DE between LF and HF groups. The R Pheatmap (1.0.8) software package was used for bidirectional cluster analysis of lncRNA and mRNA.

### 2.7. Target gene prediction and functional enrichment analysis

Gene transcript within 100 kb upstream or downstream of the DE lncRNAs was selected as cis target genes. To understand the potential roles of DE lncRNAs and DE mRNAs between LF and HF groups, Gene ontology (GO) enrichment analysis and the Kyoto encyclopedia of genes and genomes (KEGG) pathway analysis were performed to investigate the biological function of the DE lncRNAs and DE mRNAs using DAVID (http://david.abcc.ncifcrf.gov/). *p* < 0.05 indicated statistical significance.

### 2.8. LncRNA-mRNA co-expression network analysis

The Pearson correlation test was used to calculate the correlation coefficient between DE lncRNAs and DE mRNAs in the hypothalamus. The Pearson correlation coefficient >0.95 and *P-*value < 0.05 were considered statistically significant. And the data was analyzed by Cytoscape 3.9.1 to visualize lncRNA-mRNA co-expression networks.

### 2.9. qRT-PCR verification

Three DElncRNAs and three DEmRNAs were randomly selected from hypothalamic tissues, and the accuracy of RNA-Seq was verified by qRT-PCR. Total RNA was reverse transcribed to cDNA using the PrimeScript RT kit and gDNA Eraser (TaKaRa, Guangzhou, China), and design primers for lncRNA and mRNA by Primer Premier 5.0. Then the qPCR reaction was performed in triplicate using an SYBR Premix Ex Taq TM (TaKaRa, Guangzhou, China) on the Thermo Scientific StepOnePlus Real-Time System (Thermo Scientific, NY, USA) as follows: initial denaturation at 95°C for 30 s, followed by 40 cycles of 95°C for 5 s and 60°C for 30 s, respectively. Using β-actin as endogenous control, the relative expression of DE lncRNAs and DE mRNAs were quantified using the 2^−ΔΔCt^ method.

## 3. Results

### 3.1. Quality control of RNA sequencing data and read mapping

The raw reads from the high and low fecundity groups were analyzed for quality control before further analyses. In this study, 11 cDNA libraries in the hypothalamus-related tissues were constructed to identify the lncRNAs and mRNAs expressed in the HF and LF groups. The number of raw reads generated on the Illumina HiSeq platform was in the range of 101,379,018 to 106,945,128 (Supplementary Table 1). After discarding the reads containing adapter or poly-N and other low-quality reads, more than 99.10% of raw reads were filtered as clean reads and were used for the transcriptome assembly. The number of clean reads mapped to the CZ goats genome range from 86,849,768 to 92,341,252 in all libraries, and more than 97.62% were uniquely mapped. Additionally, the proportion of nucleotides with Q30 of each sample was greater than 92%. The detailed information about the genome distribution of the CZ goats genome was present in Supplementary Table 1.

### 3.2. Identification of DEmRNAs and DElncRNA in the hypothalamus of the high and low fecundity groups

In order to further explore the important regulators of the hypothalamic-pituitary-ovarian axis associated with goat prolificacy, we screened DElncRNAs and DEmRNAs between LF group and HF group according to |log2 Fold Change| > 1 and *P* value < 0.05. Consequently, a total of 23 DE lncRNA of which 14 lncRNA were up-regulated and 9 lncRNA were down-regulated were identified from the hypothalamus of HF groups compared to the hypothalamus of LF groups. The 9 down-regulated LncRNAs mainly include MSTRG.12755.2, MSTRG.20658.2 and ENSCHIT00000001669, and the 14 up-regulated LncRNAs mainly include ENSCHIT00000001894, ENSCHIT00000000939, and ENSCHIT00000009853, etc. Meanwhile, the analysis identified 57 DE mRNAs of which 27 mRNAs were up-regulated and 30 mRNAs were down-regulated between comparison groups in the hypothalamus. The 27 up-regulated mRNAs mainly included NUPR1, WT1, TP53I3 and FBLN7, and the 30 down-regulated mRNAs mainly included GNRH1, FEZF1, NPTX2, and CYP4X1 (Supplementary Table 1).

**Figure 1:**
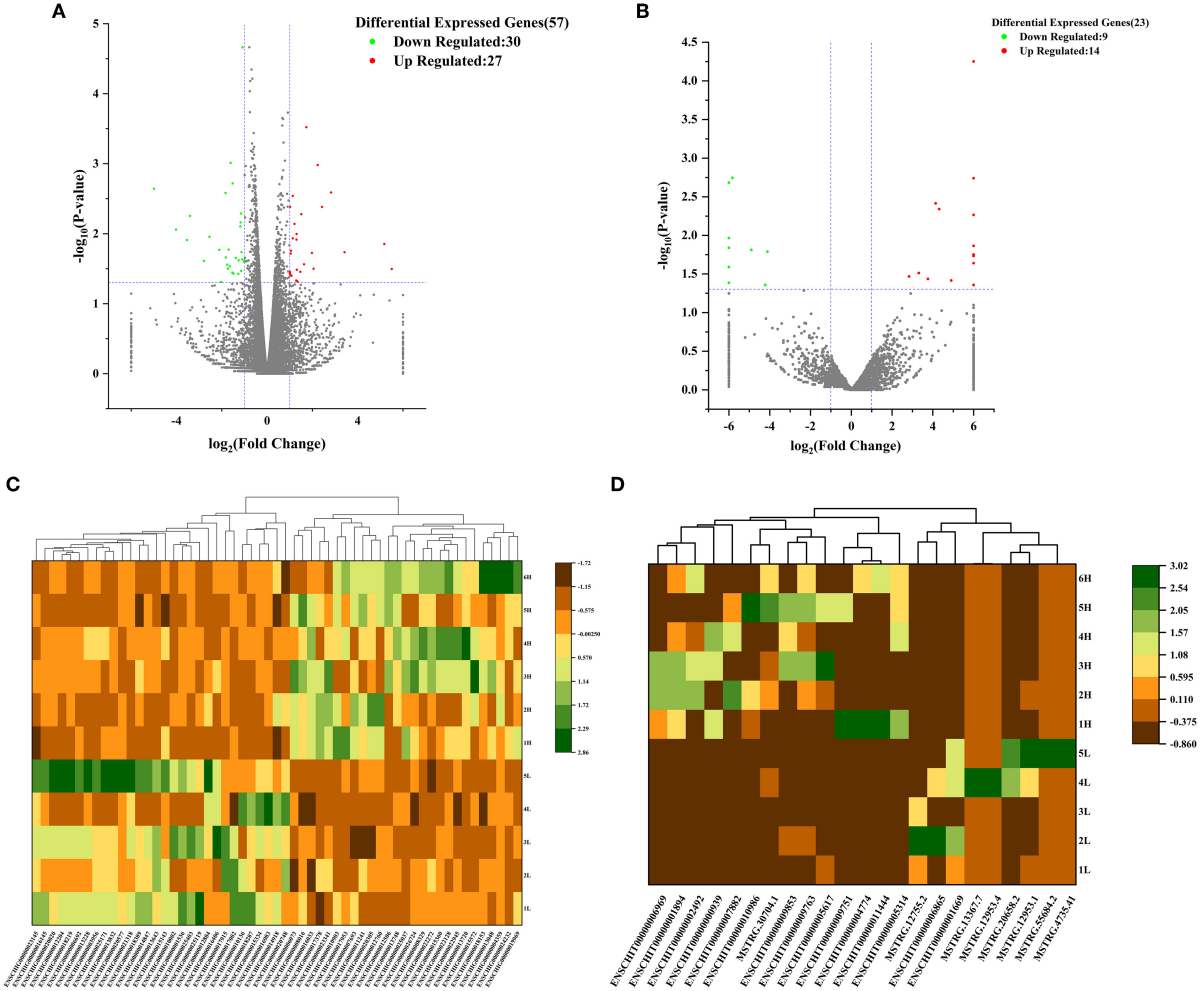
Volcano analysis of **(A)** mRNA and **(B)** lncRNA between HFG and LFG in the hypothalamus; Cluster heat map of **(C)** DEmRNAs and **(D)** DElncRNAs between HFG and LFG in the hypothalamus.

In addition, the volcano plot displayed the basic distribution of DE lncRNA and DE mRNAs, respectively (Figures 1A, B); Hierarchical cluster analysis was also used to determine the expression patterns of DE lncRNA and DE mRNAs under LF and HF conditions, and the heat maps of DE lncRNA and DE mRNAs showed a clear separation between LF and HF groups in the hypothalamus tissues, respectiveiy (Figures 1C, D). The 6 samples (H1-H6) of the hypothalamus high fecundity group were clustered in the same cluster, and the 5 samples (L1-L5) of the low fecundity group were clustered in the same cluster. The results showed that the gene expression levels and patterns of the 6 samples in the high fecundity group were similar, and the gene expression levels and patterns of the 5 samples in the low fecundity group were similar. There were significant differences between the two groups of data, which confirmed the high reliability of the hypothalamic sequencing data.

### 3.3. Functional enrichment analysis in the hypothalamus

#### 3.3.1. Functional enrichment analysis of DE lncRNAs

GO and KEGG enrichment analysis of DE lncRNAs expression was performed and found that 23 potential target genes were identified in the hypothalamus. For these genes, a total of 238 GO terms were significantly (*P* < 0.05) enriched in the hypothalamus. Of which biological processes, cellular components and molecular functions account for 188, 27, and 23 terms, respectively. And 5 KEGG pathways were enriched in the hypothalamus tissue (Supplementary Table 1).

**Figure 2:**
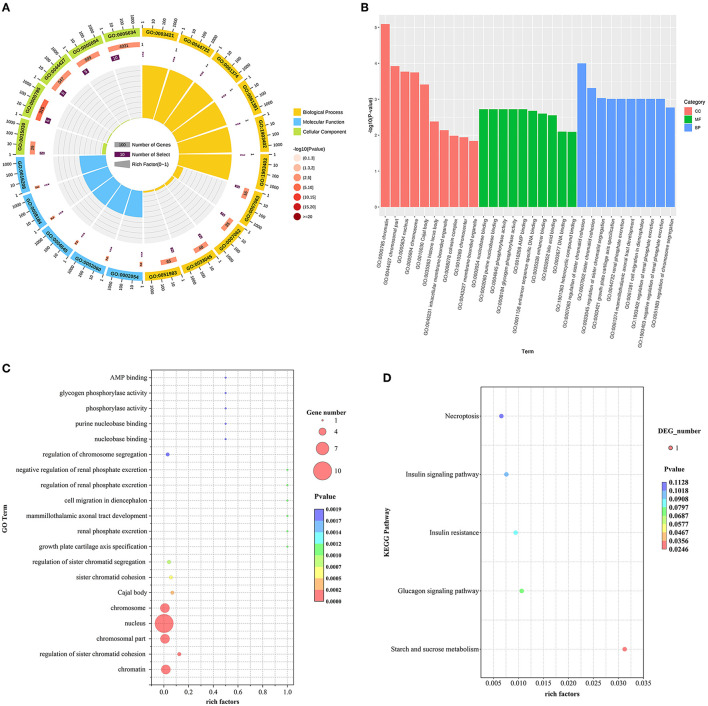
**(A–D)** GO and KEGG analyses of DE lncRNAs between HFG and LFG in the hypothalamus.

We selected the GO terms with *p* < 0.05 as a significant condition in the database, and sorted the top 20 GO terms and drew them into a bubble chart. Among them, 10 GO terms belong to biological processes, and 5 terms each for cellular components and molecular functions. Therefore, In the hypothalamus, the GO enrichment analysis showed most target genes of DE lncRNAs were mainly significantly (*P* < 0.05) enriched in nucleus, chromosomal part, chromatin, chromosome, and regulation of sister chromatid cohesion. However, Starch and sucrose metabolism were the only pathway in KEGG pathway analysis of DE lncRNAs in the hypothalamus (*P* < 0.05) (Figures 2A–D).

#### 3.3.2. Functional enrichment analysis of DE mRNAs

**Figure 3:**
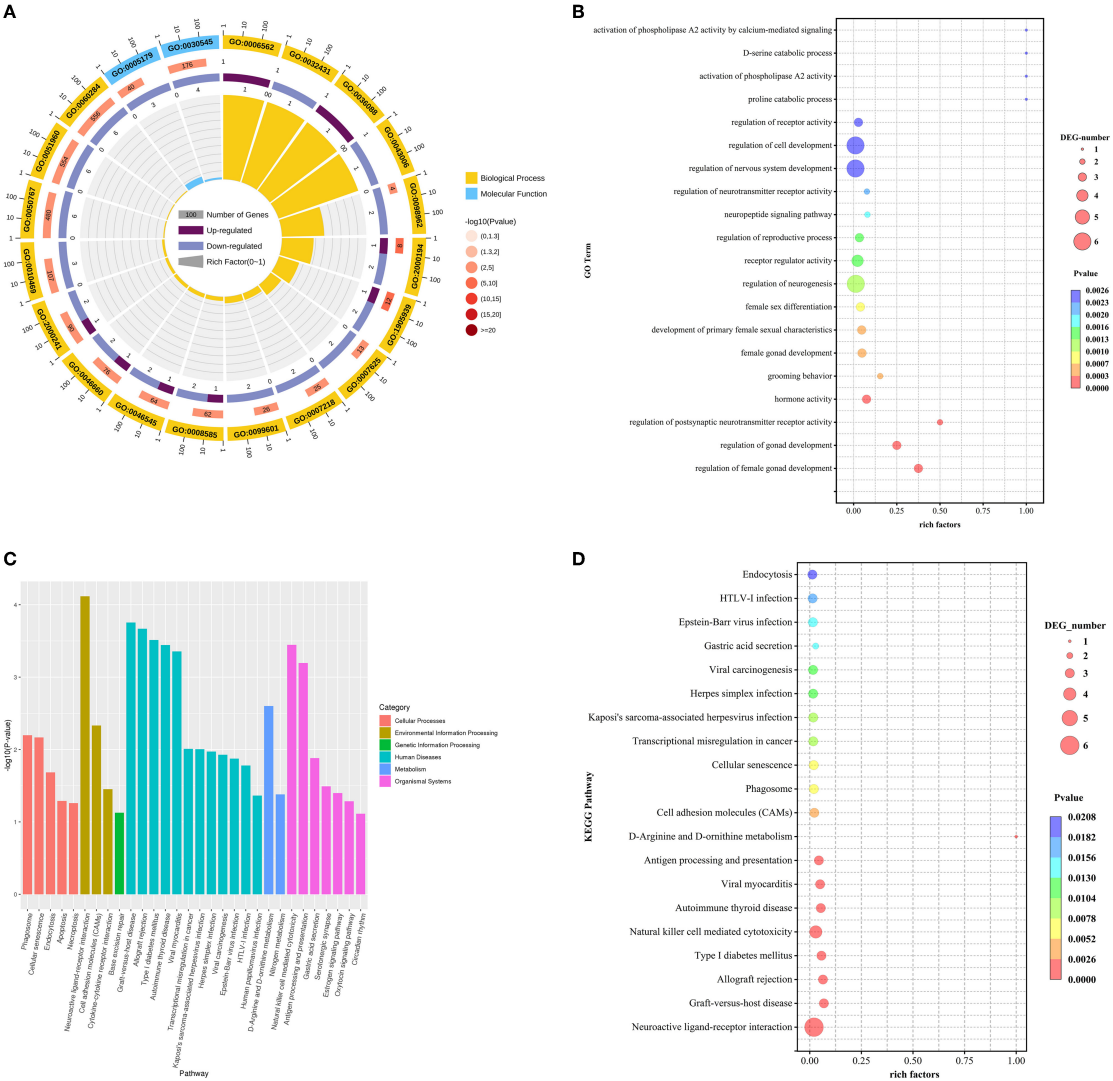
**(A–D)** GO and KEGG analyses of DE mRNAs between HFG and LFG in the hypothalamus.

For DE mRNAs, a total of 57 hypothalamic DE mRNAs were significantly enriched in 301 GO terms (*P* < 0.05). Biological processes, cellular component, and molecular functions were occupied by 256, 1, and 44 terms, respectively. The top 20 GO terms were drawn into a bubble chart, and the results showed that there are 2 terms in molecular function, mainly hormone activity and receptor regulator activity. The remaining 18 terms belong to biological processes. Among them, 32 DEGs were specifically enriched in regulation of nervous system development, receptor regulator activity, neuropeptide signaling pathway and activation of phospholipase A2 activity by calcium-mediated signaling terms, such as the down-regulated genes GNRH1, NPTX1, NPTX2, VAX1, TNFRSF12A, and SST, suggesting that these genes may be involved in the regulation of nervous system and neurotransmitter receptor activity as well as the function of synaptic signaling. Secondly, 22 DEGs were mainly enriched in terms of regulation of gonad development, hormone activity, and regulation of reproductive process, including the upregulated gene NUPR1 and the downregulated genes GNRH1, NR5A1, SST, QRFP, and VIP, indicating that these genes are mainly associated with the fecundity of goats. Finally, there are 3 DEGs mainly enriched in terms of proline catabolic process and D-serine catabolic process, such as NMUR2 and DAO, which are mainly involved in cellular metabolic pathways (Figures 3A–D).

Besides, to further clarify the contribution of specific signaling pathways to goat fecundity, analysis of KEGG pathways in DEmRNAs from high-and low-fecundity Goats revealed 25 enriched pathways, which involved Neuroactive ligand-receptor interaction, Cell adhesion molecules (CAMs), Estrogen signaling pathway, Oxytocin signaling pathway and GnRH signaling pathway (*P* < 0.05). Among them, the Estrogen signaling pathway, the Oxytocin signaling pathway and the GnRH signaling pathway were the main KEGG pathways enriched in the hypothalamus. The genes regulating the Estrogen signaling pathway were mainly the down-regulated genes KCNJ5 and MMP9. The genes corresponding to the Oxytocin signaling pathway mainly included the up-regulated gene PRKAG3 and the down-regulated gene KCNJ5. The only gene that regulates the GnRH signaling pathway is GNRH1. The top 20 GO terms and top 20 KEGG pathways for the DEmRNAs are shown in Supplementary Table 1.

### 3.4. Construction of lncRNA-mRNA co-expression network

**Figure 4:**
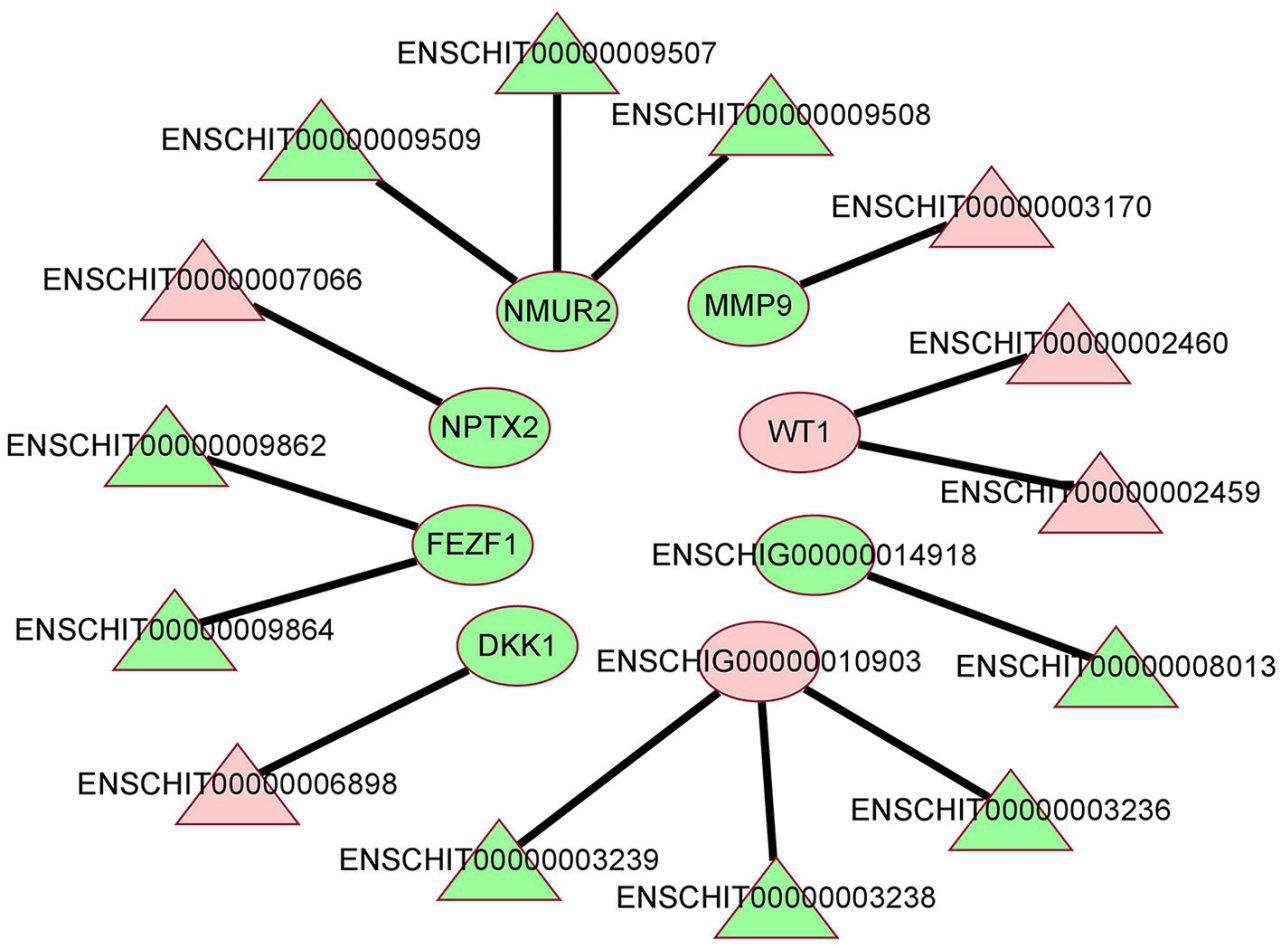
The co-expression network of lncRNA and mRNA between HFG and LFG in the hypothalamus. The red color represents upregulated expression, the green represents downregulated expression, the triangle represents lncRNA, and the ellipse represents mRNA.

To further explore the interaction between DE lncRNAs and DE mRNAs in the hypothalamus of high and low fecudity goats, we constructed network of co-expression of DE lncRNAs and DE target mRNAs. A total of 14 DE lncRNAs and 8 DE mRNAs were involved in the network, which consists of 14 edges (Figure 4, co-expression network diagram). Interestingly, 4 DE mRNAs were also found to interact with more than one DE lncRNA in the hypothalamus. The mRNAs with the largest number of interactions were NMUR2 and ENSCHIG00000010903. ENSCHIG00000010903 was negatively correlated with 3 different DE lncRNAs, while NMUR2 was positively correlated with 3 different DE lncRNAs.

### 3.5. The qPCR validation

**Figure 5:**
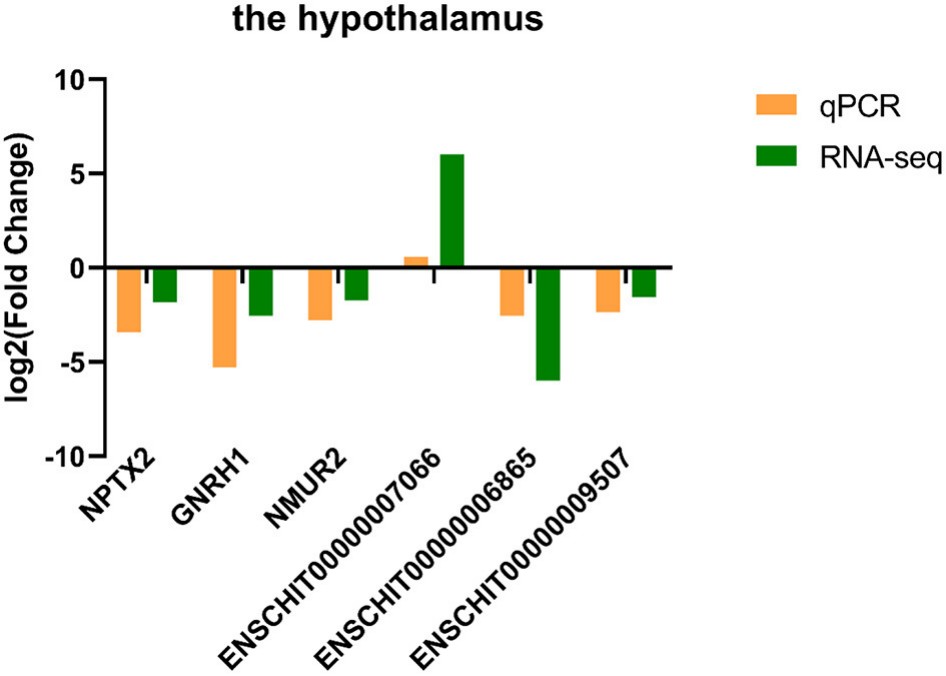
The qPCR validation results of DE lncRNAs and DE mRNAs for the hypothalamus.

Three DE lncRNAs and three DE mRNAs were randomly selected from the hypothalamus tissue for qPCR to verify their differential expression (Figure 5). The results were in concordance with the sequencing data.

## 4. Discussion

The reproductive performance of goats directly determines the productivity of animal husbandry (21). As the higher center of the neuroendocrine system, the hypothalamus releases hormones that play an indispensable role in the regulation of estrus and follicular development in goats (22). For example, it can regulate the release of E2 and P4 from the ovary by releasing gonadotropin-releasing hormone (GnRH), while acting as a facilitator or inhibitor of FSH and LH secreted by the pituitary (23). It has been shown that lncRNAs have an increasingly prominent role in reproduction in goats and can be involved in a variety of reproductive activities, such as spermatogenesis (24), placental formation (25, 26), sex hormone response signaling pathways (27), and gonadogenesis (28). However, current studies on lncRNAs associated with fecundity in goats have mainly focused on ovarian tissues (11), ignoring the importance of hypothalamic tissues. Therefore, this study investigated the expression profiles of reproduction-related lncRNAs and mRNAs in goat hypothalamic tissues by means of RNA-Seq technology, and explored the relationship between DElncRNAs and mRNAs by generating co-expression networks, thus laying the foundation for a clear explanation of the expression and regulation of reproductive traits in goats.

In this study, we selected six high-breeding and five low-breeding healthy Chuanzhong female goats for identification and characterization of lncRNAs and mRNAs in their hypothalamic tissues to identify differential mRNAs, lncRNAs and pathways associated with reproductive performance. Finally, we screened 23 DElncRNA transcripts and 57 DEmRNAs in the hypothalamus of Chuanzhong Black Goats between high and low yielding goats. In total 57 of these DEGs were mainly enriched in the regulation of ovarian development, hormone synthesis and secretion, regulation of postsynaptic neurotransmitter receptor activity and regulation of reproductive processes. NUPR, GNRH1, NR5A1, SST, QRFP, and VIP DEGs were found to be involved in the release of hypothalamic hormones and thus could regulate reproductive performance in goats. An et al. (29) analyzed 641 goats of three breeds for *GNRH1* gene polymorphism and found that GNRH1 was closely related to litter size in different breeds by association analysis and could be used as a genetic marker for litter size in goat breeding. Knight et al. (30) induced the release of LH and thus normalized estrus and ovulation in goats with estrus difficulties by injecting the exogenous hormone GnRH. KEGG enrichment analysis showed that DEGs in the hypothalamus of high and low breeding goats were mainly involved in signaling pathways such as estrogen signaling pathway, oxytocin signaling pathway and GnRH signaling pathway. Among them, DEGs such as CALM3, GNRH1, ITPR2, ADCY4, PLD2, and MAP2K1 were significantly enriched in the GnRH signaling pathway, and these DEGs may affect the kidding power of goats. Early studies have shown that calcium plays an important role in the activation and regulation of signal transduction pathways in oogenesis and that the calcium signaling pathway is a key regulatory component of meiosis during oogenesis (31). Recent studies suggest that CALM3 (calcium-modulated protein 3) may regulate oogenesis in tetraploid crucian carp through a calcium-associated pathway (32). ITPR2 is an important regulator of calcium channel activity, which is the basis of fertilization and embryonic development in animals (33, 34), and has been found to be involved in several reproduction-related pathways in sheep (35). In addition, DEGs such as NPR2, CALM3, CACNG2, KCNJ5, ITPR2, ADCY4, MAP2K1, CACNB1, and CAMK2B were mainly enriched in the oxytocin signaling pathway. C-type natriuretic peptides (NPPC) in NPPC/NPR2 signaling and its high affinity receptor natriuretic peptide receptor 2 (NPR2) are considered to be related to female reproduction (36). They act as local factors by binding to NPR2, and then produce intracellular cGMP (37), cGMP through the guanylate cyclase catalytic domain of NPR2 to regulate oocyte meiosis arrest and ovarian follicular survival (38, 39). It can be seen that NPPC/NPR2 signal transduction is very important for oocyte meiosis arrest and cumulus formation, and cumulus can affect female fertility by producing developmental oocytes (36).

The co-expression analysis of lncRNA-mRNA showed that there was a certain interaction between lncRNA and mRNA. By regulating the expression of lncRNA, the mRNA related to the above signal pathways and biological processes could be regulated to affect the fecundity of goats. A total of 14 pairs of interaction between DElncRNAs and mRNAs were found in the hypothalamus of high and low breeding goats, some of which can directly or indirectly reflect the relationship between hypothalamus and goat fecundity. NMUR2, which is down-regulated in the hypothalamus, is the predictive target gene of DElncRNAENSCHIT00000009507, ENSCHIT00000009508, and ENSCHIT00000009509. The translated protein can form a new autocrine system in membrane/interstitial cells after binding to its ligand, which plays a certain role in the ovary directly through cAMP signal transduction, in which the signal intensity is strictly controlled by gonadotropin (40). Romanov et al. (41) detected 62 neuronal subtypes in the hypothalamus by single cell RNA sequencing. The dopamine neuron gene of NMUR2 was placed in the periventricular nucleus with many synaptic afferents. It was found that these neuroendocrine dopamine cells may contribute to the inhibition of dopaminergic secretion of prolactin during the day and night. Helfer et al. (42) injected NMUR2 into the hypothalamus by making the ligand NMU of NMU partially mimic the function of thyroid stimulating hormone (TSH), which can negatively regulate food intake and body weight of rats. At the same time, NMU increased the expression of Dio2mRNA in the ependymal area of the hypothalamus. These results suggest that NMUR2 may affect the release of reproduction-related hormones in the neuroendocrine system and hypothalamus in different ways. In addition, DE lncRNA ENSCHIT00000009862 and ENSCHIT00000009864 in the hypothalamus are predicted to act on the target gene FEZF1, a family of Fez transcription factors that control neurogenesis and cellular fate in the developing mammalian nervous system (43). Zinc finger transcription factor FEZF1 is a highly conserved family of transcription factors that play a role in neurogenesis, developmental patterns, cell fate regulation and axonal guidance (44). And the function of the down-regulated target gene FEZF1 is consistent with the corresponding regulation of neurogenesis (GO:0050767) GO term in our sequencing results. In addition, we also found that the target gene WT1 corresponding to up-regulated DE lncRNA ENSCHIT00000002459 and ENSCHIT00000002460 was also closely related to ovarian follicular development in female animals. WT1 is a nuclear transcription factor, and Chen et al. (45) found that protein arginine methyltransferase 5 (PRMT5) deficient mouse follicular granulosa cells expressing steroid genes were reversed by WT1 overexpression, indicating that PRMT5 is necessary to prevent premature differentiation of granulosa cells by regulating WT1 expression. There is also a down-regulated DElncRNAENSCHIT00000003170 predicted target gene MMP9, as a matrix metalloproteinase regulated by LH or progesterone, which not only affects cell growth, differentiation, proliferation and apoptosis, but also plays an important role in ovulation and gonadal formation in female animals (46). Shah et al. (47) found that MMP9 is a key enzyme to maintain the normal physiology of the female reproductive system, and it can also participate in neuroendocrine regulation through gonadotropin-releasing hormone (GnRH). GnRH-mediated ERK1/2 activation in hypothalamic neurons and pituitary gonadotropins depends on MMP9. In addition, cell migration, division, differentiation, survival or death requires extracellular matrix (ECM) to provide the environment, and ECM homeostasis is strictly regulated by MMP9.

In conclusion, as the primary site in HPOA, the key function of the hypothalamus is to precisely regulate follicular development in goats by releasing hormones (48). The DElncRNAs identified in this study cooperate with their target genes and DEGs to regulate hypothalamic function and reproductive processes through hormones and other regulators. However, we provided few indirect experimental results to infer the functional connectivity between lncRNA-mRNA network components, thus preventing a more complete and definitive proof of the obtained results, suggesting that there are still some limitations in our present study. In future studies, we will further demonstrate the linkage of our predicted lncRNAs and their potential target genes and elucidate how these differential lncRNAs and mRNAs play a role in goat fertility.

## 5. Conclusions

In this study, we revealed the regulation of differential lncRNAs and mRNAs in pathways associated with reproductive function in high- and low-fecundity goats through hypothalamic transcriptomics. Using the low-breeding group as a control, mRNA and lncRNA from the hypothalamus of goats in the high-breeding group were screened for differences and identified, and DElncRNA and DEG were enriched and analyzed, And we also construct the interaction network between lncRNA and mRNA to provide some ideas for the follow-up study of the regulation mechanism of functional lncRNA in hypothalamus on goat follicular development.

## Data availability statement

The original contributions presented in the study are included in the article/Supplementary material, further inquiries can be directed to the corresponding authors.

## Ethics statement

The animal study was reviewed and approved by Ethics Committees of the Laboratory Animal Center of South China Agricultural University (permit number: SYXK-2014-0136). Written informed consent was obtained from the owners for the participation of their animals in this study.

## Author contributions

Sample collection: SD and MM. Data curation and writing—original draft: BH. Methodology: BH and MM. Project administration: GL and DL. Software: GL and BS. Supervision: GL and YL. Validation: BH and SD. Writing—review and editing: GL. All authors have read and approved the manuscript.
